# Programmatic Impact of QuantiFERON-TB Gold In-Tube Implementation on Latent Tuberculosis Diagnosis and Treatment in a Public Health Clinic

**DOI:** 10.1371/journal.pone.0036551

**Published:** 2012-05-07

**Authors:** Maunank Shah, Danielle DiPietro, Adena Greenbaum, Sherry Ketemepi, Maria Martins-Evora, Vincent Marsiglia, Susan E. Dorman

**Affiliations:** 1 Johns Hopkins University School of Medicine, Baltimore, Maryland, United States of America; 2 Baltimore City Health Department, Baltimore, Maryland, United States of America; 3 Tulane University, New Orleans, Louisiana, United States of America; McGill University, Canada

## Abstract

**Background:**

QuantiFERON-TB Gold In-Tube (QFT-GIT) is considered an alternative to the tuberculin skin test (TST) for the diagnosis of tuberculosis (TB) infection, but the programmatic impact of QFT-GIT implementation is largely unknown. In March, 2010, the Baltimore City Health Department (BCHD) introduced routine QFT-GIT testing for individuals referred to the TB program for suspected latent TB infection (LTBI).

**Design:**

Retrospective study comparing LTBI diagnosis and treatment during the 13 months before and after QFT-GIT implementation at the BCHD TB clinic.

**Results:**

607 and 750 individuals were referred by community-providers for suspected LTBI in the pre- and post-QFT-GIT periods, respectively. Most individuals in the pre- and post-QFT-GIT periods were referred on the basis of a positive TST (597/607 [98%] vs. 690/750 [92%], respectively) and were foreign-born (363/607[59%] vs. 507/750[68%], respectively). BCHD performed QFT-GIT testing for 375/543 (69%) eligible individuals in the post-QFT-GIT period, of which 185 (49%) were positive, 178 (47%) were negative, 1 (0.25%) was indeterminate, and 11 (3%) did not yield results. Concordance of QFT-GIT with TST was low (183/352[52%]). Foreign-born individuals had higher proportions of QFT-GIT positivity (57%) than US-born individuals (36%; AOR 3.3 [95%CI 1.7–6.2]). Significantly fewer individuals received a final diagnosis of LTBI in the post-QFT-GIT period (397/567 [70%]) compared to the pre-QFT-GIT period (445/452 [98%], p<0.001). In the post-QFT-GIT period, only 230/399 (58%) of those receiving QFT-GIT testing had a final diagnosis of LTBI, while 167/168 (99%) of those without QFT-GIT testing were diagnosed with LTBI (p<0.001). There was no difference in treatment initiation between those with and without QFT-GIT testing (175/230 [76%]) vs. 133/167 [80%], respectively) in the post-QFT-GIT period.

**Conclusion:**

QFT-GIT implementation for LTBI evaluation in a public health clinic significantly reduced the proportion of referred individuals in whom LTBI was diagnosed. QFT-GIT testing had no impact on treatment initiation or completion among those diagnosed with LTBI.

## Introduction

Testing and treatment of persons at increased risk for latent tuberculosis infection (LTBI) is a core element of the tuberculosis (TB) elimination strategy in the United States (US) [1], [2], [3]. The tuberculin skin test (TST) is widely utilized for detection of *M. tuberculosis* infection, but this test has important limitations. The TST can cross-react with non-tuberculous mycobacterial (NTM) species or Bacille Calmette Guerin (BCG) vaccine, thereby complicating the interpretation of TST results especially in BCG-vaccinated foreign-born individuals from TB-endemic settings. Additionally, TST results are subject to inter-reader variability and may differ by level of training of those reading the test [4]. These limitations may reduce TST specificity, and may reduce patient and provider confidence in TST results.

Interferon-gamma (IFN-γ) release assays (IGRAs), such as the commercially available QuantiFERON-TB Gold In Tube (QFT-GIT, Cellestis, Ltd, Carnegie, Australia) test, have the potential to overcome some of TST's limitations. QFT-GIT detects *M. tuberculosis* (MTB) infection by measuring *in vitro* IFN-γ release following stimulation of lymphocytes with antigens specific to *M. tuberculosis,* ands has several potential advantages over TST. QFT-GIT retains specificity in BCG-vaccinated populations and has less cross-reactivity than TST with NTM species [5], [6], [7]. QFT-GIT has similar or potentially increased sensitivity compared with TST based on a recent meta-analyses of the literature [6]. Since QFT-GIT is a quantitative blood test, its results are less subjective than those of TST. Recently the U.S. Centers for Disease Control and Prevention (CDC) provided guidance that IGRAs are an acceptable alternative to TST for the detection of *M. tuberculosis* infection, and are the preferred option in some circumstances including testing of BCG-vaccinated populations [7].

While many research studies have been conducted to assess IGRA test performance, there is limited information about the implementation of these tests in the context of public health TB control programs [8], [9], [10]. Results from a limited San Francisco Department of Public Health program to substitute TST with QFT-GIT suggested that QFT-GIT testing was feasible with results more readily available than TST results [8]. In Alberta, Canada QFT-GIT was used as a confirmatory test for patients with a positive TST who were referred to a TB clinic. They found only 40% of patients referred for a positive TST were QFT-GIT positive, suggesting possible high proportions of false-positive TST results due to BCG vaccination or misreading of TSTs [9]. Whether QFT-GIT can be successfully implemented into other local health department TB programs, and the ultimate programmatic impact on LTBI diagnosis and treatment remains unclear.

The Baltimore City Health Department (BCHD) TB control program clinic (“BCHD TB clinic”) provides TB clinical care services free-of-charge to residents of Baltimore City, population 620,961, with active TB incidence of 5.2/100,000 in 2010[11]. With respect to *M. tuberculosis* infection, a main BCHD TB clinic activity is the evaluation and clinical management of individuals with suspected *M. tuberculosis* infection referred from community providers. Within Baltimore City, a variety of clinical care providers perform *M. tuberculosis* infection testing, almost exclusively using TST, for a number of indications including employment testing, immigration/refugee services, homeless services, and as a requirement for attendance in some drug-treatment programs. Individuals identified with possible *M. tuberculosis* infection (typically individuals with a positive TST) by community sources are referred to the BCHD TB clinic for further evaluation and treatment. Prior to March 2010, the BCHD TB clinic made decisions on LTBI diagnosis and treatment based upon test results (i.e. TST in most cases) available from the referral source. In March 2010, the BCHD TB clinic decided to implement a new testing program in which QFT-GIT was made available as part of normative clinical care during evaluations for individuals referred with suspected *M. tuberculosis* infection. We sought to formally evaluate the programmatic impact of QFT-GIT implementation in the BCHD TB program. We assessed the uptake of QFT-GIT testing and availability of interpretable results, and compared programmatic rates of LTBI diagnosis and treatment before and after QFT-GIT implementation. Additional analyses attempted to determine factors associated with QFT-GIT test positivity and concordance with TST.

## Methods

### Ethics Statement

This retrospective study was approved by ethics committees at the Johns Hopkins University School of Medicine (Baltimore, USA) and the Baltimore City Health Department. This study received a waiver of informed consent; this research involved no more than minimal risk to subjects, data was collected solely by review of existing laboratory and medical records, and this research could not practicably be carried out without the waiver of informed consent.

### QFT-GIT Implementation

The BCHD TB clinic initiated QFT-GIT testing on March 1, 2010 as part of their normative care algorithms. Following March 2010, staff routinely obtain blood for QFT-GIT testing on all individuals referred for suspected *M. tuberculosis* infection who had not had prior QFT-GIT testing from the referral source. Clinical care staff underwent manufacturer-supervised training in filling the tubes during phlebotomy, and in how to label and send tubes to the single off-site laboratory located approximately 3 miles away prior to QFT-GIT implementation into routine practice. The four prescribing clinicians (2 MDs and 2 Nurse Practitioners) were trained in interpretation of QFT-GIT test results and CDC recommendations [7]. Manufacturer representatives trained laboratory personnel in the correct performance of the assay. Within the BCHD TB clinic no incubator was available, and tubes are transported once per day at approximately 2 pm from the clinic to the testing laboratory. Within BCHD TB clinic, clinicians are allowed to use clinical judgment and not send QFT-GIT test if blood cannot be readily obtained from an individual, or if the specimen transport courier has already completed specimen pick-up for the day.

### Study Design

We conducted a retrospective cohort study to evaluate LTBI services before and after implementation of the QFT-GIT test in the BCHD TB program, and assessed concordance of QFT-GIT with TST during routine clinic conditions. Data was obtained using the BCHD TB Program electronic database (Microsoft Access 2003) and through chart reviews. We compared 13 months of data prior to QFT-GIT introduction (pre-QFT-GIT) with the 13 month period after QFT-GIT implementation (post-QFT-GIT). Patients were assigned to the pre-QFT-GIT period if they were referred or evaluated between Feb 1, 2009 and Feb 28, 2010; patients were assigned to the post-QFT-GIT period if they were referred or evaluated between March 1, 2010 and March 31, 2011.

### Study Population

Individuals referred to the BCHD TB clinic for suspected *M. tuberculosis* infection were included without age restriction. Individuals are referred to BCHD for evaluation on the basis of a positive TST, positive IGRA test, or an immigration B-Waiver (individuals with evidence of inactive TB infection on chest radiographs at the time of immigration). Individuals with active TB and their close contacts were excluded.

### LTBI evaluation at BCHD

Per routine care, all individuals referred for *M. tuberculosis* infection evaluation were interviewed by a BCHD TB clinic staff member for demographic information, medical history, and signs and symptoms of active TB; a chest x-ray and liver chemistries were obtained, and HIV testing was offered. TST and QFT-GIT were not repeated for individuals who had one or both tests performed by a community provider. Patients with signs or symptoms of active TB were evaluated further by sputum smear microscopy, culture, and other testing as indicated.

### Latent TB diagnosis and treatment

Prior to March 1, 2010, diagnosis of LTBI was based on available TST or IGRA information from the referral source at the time of referral to the BCHD TB clinic. Starting March 1, 2010, BCHD directed QFT-GIT testing was made available as part of the diagnostic evaluation. Individuals with discordant results on TST and QFT-GIT could be diagnosed with LTBI at the discretion of the BCHD TB clinician. Factors considered by clinicians included but were not limited to HIV status, chest x-ray results, age of individual, degree of TST induration, and QGIT antigen and nil results, country of origin, BCG status, and other TB risk factors. Individuals diagnosed with LTBI during both study periods were offered treatment in accordance with published guidelines, with medications dispensed on a monthly basis after monthly follow-up BCHD TB clinic visits including toxicity assessment [12], [13].

### QFT-GIT testing

QFT-GIT testing was performed according to manufacturer's instructions at a single off-site BCHD laboratory [14]. Phlebotomy for QFT-GIT testing occurred at the BCHD TB clinic at the time of the patient's initial clinical evaluation. Samples were stored at room temperature for up to 6 hours at the BCHD TB clinic until transportation to the laboratory via a daily courier service. Following incubation and centrifugation, harvested plasmas were stored at 4°C for up to 17 days prior to ELISA testing. Results were calculated and interpreted by the assay software as positive, negative, or indeterminate, according to manufacturer's instructions– tests were interpreted as indeterminate if the Mitogen minus Nil was <0.5, or the Nil was >8.0; tests were interpreted as negative if the TB antigen minus Nil was <0.35, or if the TB antigen minus nil was ≥0.35 but was <25% of the Nil value; tests were interpreted as positive if the TB Antigen minus Nil was ≥0.35 and was ≥25% of the Nil value [14].

### Statistical Considerations

The primary objectives of this study were to determine the proportions of LTBI diagnosis and subsequent treatment initiation among individuals referred to BCHD for TB evaluation,comparing the pre-QFT-GIT and post-QFT-GIT period. Additional analysis sought to determine proportions of QFT-GIT positivity among those referred to BCHD for LTBI care, to assess the percent agreement between TST and QFT-GIT, and to assess factors associated with QFT-GIT positivity and concordance with TST. Categorical data were compared using χ^2^ tests. Factors associated with QFT-GIT results and TST concordance were assessed using univariate and multivariate logistic regression analysis. Analysis of treatment completion was restricted to those who started a 9 month INH regimen prior to Nov 30, 2010 or a 4 month Rifampin regimen prior to March 30, 2011, to allow time for treatment completion. Data were analyzed using STATA (version 10.1, StataCorp, College Station, Texas).

## Results

### Study Population

**Figure 1:**
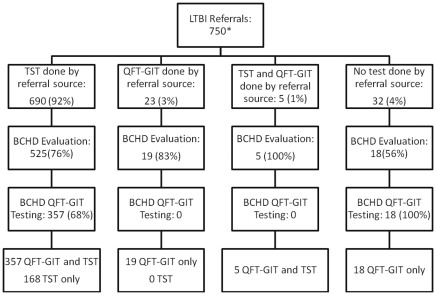
Flow of LTBI patient evaluation and testing at BCHD in the post-QFT-GIT period. *Among 750 referrals, 690 had a TST by referring source, 23 had a QFT-GIT by the referring source, and 5 had both a QFT-GIT and TST performed by referral source); 32 individuals were referred as B-Waivers without prior LTBI testing based on abnormal CXR during immigration. Among 567 referrals that came to BCHD for evaluation, 525 were referred with a TST result and 19 for a positive QFT-GIT and 5 individuals had both a QFT-GIT and TST performed by referral source; 18 B-waiver referrals were evaluated without prior LTBI testing. Among these 567 referrals, 168 had only a TST (30%), 37 had only a QFT-GIT (67), and 362 (64%) had both a TST and a QFT-GIT test result. Of 399 QFT-GIT test results among referrals evaluated by BCHD, 375 had QFT-GIT testing at BCHD and 24 had QFT-GIT testing from referral source.

Table 1 shows demographic features of individuals referred to the BCHD TB clinic for suspected *M. tuberculosis* infection. There were more referrals in the post-QFT-GIT period (750) compared to the pre-QFT-GIT period (607, p<0.01). Among referrals, there was no difference in age, sex, or HIV status comparing the two study periods (Table 1). However, compared to the pre-QFT-GIT period, the post-QFT-GIT period had a greater proportion of individuals identified as being foreign-born (68% post-QFT-GIT compared to 59% pre-QFT-GIT, p = 0.003). The most common countries of origin among foreign-born in the pre-QFT-GIT period were from Nepal (58/363 [16%]), Bhutan (54/363 [15%]), Burma (22/363[6%]), and Iraq (21/363[6%]). In the post-QFT-GIT period the most common countries of origin for foreign-born were from Nepal (72/507[14%]), Bhutan (62/507[12%]), Mexico (40/507 [8%]), Iraq (25/507[5%]), Ethiopia (25/507[5%]) and Eritrea (25/507[5%]). The referring sources also differed slightly between the two study periods–there was a decline in the percentage of referrals from drug treatment programs and an increase in the percentage of referrals from health fairs (targeting the Latino community) and B-waivers in the post-QFT-GIT period compared to the pre-QFT-GIT period (Table 1). In the pre-QFT-GIT period, 597/607(98%) were referred on the basis of a positive TST, 3/607 (1%) for a positive QFT-GIT, and 7/607 (1%) were referred with no LTBI test (on the basis of a B-waiver). In the post-QFT-GIT period, 690/750(92%) were referred for a positive TST alone, 23/750(3%) for a positive QFT-GIT alone, 32/750 (4%) for a B-waiver with no LTBI test, and 5/750(1%) with both a QFT-GIT and TST performed (Figure 1).

**Table 1:** Characteristics of individuals referred to Baltimore City Health Department TB Clinic for evaluation of suspected *M. tuberculosis* infection, by study period.

Characteristic		Referrals		
		Pre-QFT-GIT	Post-QFT-GIT	P value
Number		607	750	
Evaluated by BCHD during in-person clinic encounter		452 (75%)	567(76%)	0.631
Gender	Female	255(42%)	325(43%)	0.624
	Male	352(58%)	425(57%)	
Age	Mean Age(SD)	36.1 (15.3)	36.4 (16.5)	0.77
Foreign-born		363(59%)	507(68%)	0.003
Ethnicity*	Black	260(43%)	296(39%)	0.002*
	Asian/Pacific Island	199(32%)	224(30%)	
	Latino	65(11%)	122(17%)	
	White	43(7%)	35(5%)	
	Other/Unavailable	40(7%)	73(10%)	
HIV †	Positive	11/452(2%)	19/567(3%)	0.599
	Negative	316/452(70%)	401/567(71%)	
	Refused/Unknown	125/452(28%)	147/567(26%)	
Referral Source:	Drug Treatment Program	134 (22%)	104(14%)	<0.001
	Refugee	194(32%)	237(32%)	0.904
	B-Waiver	25(4%)	72(10%)	<0.001
	Health Fairs	19(3%)	40(5%)	0.047
	Civil Surgeons	21(3%)	32(4%)	0.441
	HIV clinics	7(1%)	17(2%)	0.121
	Local Health Departments**	52(9%)	66(9%)	0.879
	Dept of Corrections	4(1%)	3(.5%)	0.509
	Occupational Health	14(2%)	9(1%)	0.116
	Obstetricians	20(3%)	22(3%)	0.706
	Primary Care Providers/Other	117(19%)	148(20%)	0.865

Abbreviations: SD, Standard Deviation. BCHD, Baltimore City Health Department.

* Ethnicity data was based on referral documentation and/or initial evaluation at BCHD. P-value for global comparison of equality of proportions of ethnicities by χ^2^ test.

† HIV test results are available only for those that came to BCHD for evaluation. HIV status not available for those who did not complete an LTBI evaluation at BCHD.

** Includes referrals from other local health departments in Maryland and other states, as well as employment TB testing conducted through other BCHD programs.

There was no difference in the overall number of referred individuals that came to BCHD TB clinic for their initial appointment between the two study periods (452/607 [75%] and 567/750 [76%] in the pre- and post-QFT-GIT periods, respectively; p = 0.631). Characteristics of individuals adhering to an initial BCHD TB appointment are shown in Table S1. There was no difference in age or gender among those adhering to an initial BCHD evaluation compared to those who did not come for initial evaluation within either study period. In both study periods, a significantly higher proportion of foreign-born individuals adhered to an initial BCHD evaluation compared to US born individuals (Table S1).

### Implementation of QFT-GIT

Among the 567 referrals that came to BCHD for evaluation in the post-QFT-GIT period, 525 (93%) were referred with a TST, 19 (3%) for a positive QFT-GIT, and 5 (1%) individuals had both a QFT-GIT and TST performed by referral source; 18 (3%) B-waiver referrals were evaluated without prior LTBI testing. Among these 567 referrals, 543 did not have a prior IGRA test and were eligible for QFT-GIT testing at BCHD. QFT-GIT testing was performed for 375/543 (69%) eligible individuals (Figure 1). Individuals coming to the clinic after the courier had completed daily pick-up of blood specimens were not able to have QFT-GIT testing performed; time of patient evaluation was not available and could not be further explored. There was a difference in implementation of QFT-GIT testing by age (p<0.01), with a lower proportion of younger children being tested compared to adults (0/3[0%] in ages 0–2, 3/45[7%] in ages 2–12, 23/36[64%] in ages 13–17, 258/336[77%] in ages 18–50, and 91/123[74%] in adults >50). There was also less QFT-GIT testing in individuals known to be HIV positive (9/17[53%]) compared to individuals that were HIV-negative (305/288 [79%]; p<0.01). There were no differences in the proportion tested by gender, birth country, race, or referral source (Table S2).

### Results of QFT-GIT testing

Among the 375 individuals who underwent QFT-GIT testing at BCHD TB clinic, 185 (49%) were positive by QFT-GIT, 178 (47%) were negative, and 1 (0.25%) was indeterminate, for 11 (3%) individuals the QFT-GIT test did not yield results due to specimen processing or transportation errors.

Factors associated with QFT-GIT positivity among those receiving QFT-GIT at BCHD are shown in Table 2. There were no differences in age, sex, or ethnicity with regard to QFT-GIT test positivity. Foreign-born individuals referred for LTBI evaluation had a higher proportion of QFT-GIT positivity (57%) compared to US born individuals (36%; AOR 3.3 [95%CI 1.7–6.2]; p<0.01). Among foreign-born, there were no differences in QFT-GIT positivity by geographic region of origin (Central/South America 25/51 [49%], Asia 66/115[57%], Africa 42/63 [67%], Europe 1/3 [33%], Caribbean 1/3 [33%], Other/Unspecified 11/21 [52%]; p = 0.382). There was a difference in QFT-GIT test positivity when comparing referring sources in univariate analysis (Table 2), with highest QFT-GIT positivity seen among those referred from refugee programs (92/144 [64%], OR 2.7 [95%CI 1.5–4.9]; p = 0.001) and other local health departments (22/35 [63%], OR 2.6 [95%CI 1.1-6.0]; p = 0.03). Referral source was not included in multivariate analysis due to colinearity with birth-country. We observed a trend in the relationship between QFT-GIT positivity and degree of TST induration, with increasing QFT-GIT positivity with higher amounts of TST induration, but this relationship was not statistically significant. QFT-GIT was positive in 3/8 (38%) individuals with TST between 0–10 mm, 76/184 (41%) of individuals with TST between 10–15 mm, 59/95 (62%), of those with TST between 15–20 mm, and 40/59 (68%) of those with TST>20 mm.

**Table 2:** Factors associated with QFT-GIT test positivity among those tested at BCHD.

Characteristic		Referral for LTBI		OR	AOR
		N	QFT-GIT positive(%)		
QFT-GIT performed by BCHD		375 *			
Interpretable QFT-GIT result available		363*	185 (51%)		
Gender	Female	166	81 (49%)	REF	REF
	Male	197	104 (53%)	1.2(.78–1.8)	1.2(0.79–2.0)
Age	0-2	0	–	–	–
	2-12	3	1 (33%)	0.46 (0.03–5.2)	0.11(0.01–1.5)
	13-17	23	11 (48%)	0.83(.33–2.1)	0.39(0.14–1.2)
	18-50	251	128 (51%)	0.94(.58–1.5)	0.59(0.32–1.1)
	>50	86	45 (52%)	REF	REF
Birthplace	Not Foreign-born	107	39 (36%)	REF	REF
	Foreign-born	256	146(57%)	**2.3 (1.4**–**3.7)** °	**3.3(1.7**–**6.2)**°
Ethnicity	White	18	7 (39%)	REF	REF
°	Asian/Pacific Island	123	70 (56%)	2.1 (0.75–5.7)	1.2(0.37–3.9)
°	Black	150	77(51%)	1.6 (0.61–4.5)	1.3(0.45–4.0)
°	Latino	56	26(46%)	1.4(0.47–4.0)	0.73(0.21–2.5)
°	Other/Unavailable	16	5(31%)	0.7(0.17–3.0)	0.73(0.16–3.3)
HIV	Positive	9	1(11%)	**.011 (0.01**–**0.92)** °°	0.17(0.02–1.77)
°	Negative	295	154 (52%)	REF	REF
	Unknown	59	30 (51%)	0.94 (.54–1.7)	1.6(0.80–3.1)
Referral Source:	Drug Treatment Programs	44	14 (32%)	0.70(0.3–1.6)	†
°	Refugee Services	144	92 (64%)	2.7 (1.5–4.9) ††	
	B-Waiver	34	16 (47%)	1.4 (0.58–3.1)	
	Health Fairs	18	8 (44%)	1.2(0.42–3.5)	
°	Immigration/Civil Surgeons	6	3 (50%)	1.5(0.28–8.1)	
°	HIV	8	1(13%)	0.22 (0.02–1.9)	
°	Local Health Departments	35	22(63%)	2.6 (1.1–6.0) ††	
	Dept of Corrections	1	0 (0%)	–	
	Occupational Health	4	3 (75)	4.6 (0.45–46)	
	Obstetricians	6	1 (16%)	0.3 (0.03–2.8)	
	Primary Care Providers/Other	63	25 (40%)	REF	
TST Induration	0–10mm		3(38%)	REF	REF
	10–15mm	184	76(41%)	1.2 (0.27–5.1)	0.99(0.18–5.5)
	15–20mm	95	59(62%)	2.7 (0.6–12)	2.4(0.41–13.9)
	>20mm	59	40(68%)	3.5(0.8–16)	2.8(0.47–16.7)

Only individuals with QFT-GIT performed by BCHD are included. 11 individuals had blood drawn for QFT-GIT but did not have interpretable results due to insufficient blood volume during venipuncture, sample transportation issues, or processing error. There was 1 indeterminate result.

°p<0.001 for both univariate and multivariate analysis comparing foreign-born to US born individuals.

°° P = 0.042 comparing HIV positive to HIV negative individuals.

† Referral source was omitted from multivariate regression model due to collinearity with birth country.

†† p = 0.001 comparing those referred from Refugee health services to those referred from primary care providers/other; p = 0.03 comparing those referred from local health departments to those referred from primary care providers/other.

### Concordance between TST and QFT-GIT tests

During the post-QFT-GIT period, 352 individuals referred for evaluation had both an interpretable QFT-GIT and an available TST result available in the post-QFT-GIT period (all 352 TSTs were performed by the referral source; 347 QFT-GITs were performed by BCHD and 5 QFT-GITs were performed by the referral source). Overall, there was only modest agreement between TST and QFT-GIT (183/352[52.0]%; Table 3). Among those that were referred with a positive TST result, QFT-GIT was positive in only 179/344 (52.0%) individuals. Individuals that were foreign-born were significantly less likely to have discordant results compared with those that were US-born (41% discordance versus 63% discordance; AOR 0.34 [95% CI 0.18-0.63]; p = 0.001; Table S3). Referral source was also associated with discordance in univariate analysis, with those referred from refugee services and local health department programs less likely to have discordance compared to those referred by primary care doctors or other health centers (Table S3).

**Table 3:** Concordance of TST and QFT-GIT results among referred individuals that came to BCHD for LTBI evaluation and had both tests performed.

	TST negative	TST positive	Total
**QFT-GIT negative**	4 (1%)*	164 (47%)	168
**QFT-GIT positive**	4 (1%)*	179 (51%)	183
**QFT-GIT indeterminate**	0	1 (0.25%)	1
**Total**	8	344	352†

† Overall, 352 individuals had a TST and interpretable QFT-GIT result available. There was an overall concordance of 52.3%.

* 8 individuals with negative TST results were referred and evaluated by BCHD. 4 individuals with B-waivers had negative TST, but were referred due to an abnormal CXR; 4 individuals had both TST and QFT-GIT performed by referral source.

**Table 4:** Differences in LTBI diagnosis among referrals to BCHD between study periods and by QFT-GIT test status.

Group	QFT-GIT category	Referral N	Evaluated by BCHD N (%)	Diagnosed LTBI N (%)	Initiation of treatment N (%)**	Completion of treatment†
Pre-QFT-GIT	Total	607	452 (75%)	**445 (98%)°**	341 (77%)	251 (74%)
	QFT-GIT performed		3 (1%)*	3 (100%)°°	2 (66%)	2(100%)
	• QFT-GIT negative		• –	• –	• –	• –
	• QFT-GIT positive		• 3 (100%)	• 3 (100%)	• 2 (66%)	• 2 (100%)
	No QFT-GIT performed		449 (99%)	442 (98%)°°	339 (77%)	249(78%)
Post-QFT-GIT	Total	750	567 (76%)	**397(70%)**°	307 (77%)	174/244 (75%)
	QFT-GIT performed		399 (70%)*	**230 (58%)**°°	174 (76%)	105/137(77%)
	• QFT-GIT negative		• 178 (45%)	• 10 (6%)	• 10(100%)	• 4/7(57%)
	• QFT-GIT positive		• 209 (52%)	• 209 (100%)	• 157(75%)	• 97/120(81%)
	No QFT-GIT performed		168 (30%)	**167 (99%)**°°	133 (80%)	69/107(65%)

* includes individuals that had QFT-GIT performed by referral source. 11/399 individuals in the post-QFT-GIT period had QFT-GIT drawn but no results available; there was 1 indeterminate result in the post-QFT-GIT-period.

°p<.001 comparing final diagnosis of LTBI between pre-QFT-GIT and post-QFT-GIT periods.

°°p = .827 for pre-QFT-GIT period comparing LTBI diagnosis between those with and without a QFT-GIT result; p<.001 in post-QFT-GIT period comparing LTBI diagnosis between those with and without QFT-GIT performed.

** p = .81 comparing treatment initiation among those diagnosed with LTBI between pre-QFT-GIT and post-QFT-GIT periods; p = 0.690 comparing treatment initiation between those with and without QFT-GIT performed in the pre-QFT-GIT period; p = .349 comparing treatment intiation between those with and without QFT-GIT performed in the post-QFT-GIT period.

† Analysis restricted to those who started an INH X 9 months regimen prior to Nov 30, 2010 or Rifampin X 4 months prior to March 30, 2011 to allow time for completion. p = .606 comparing overall treatment completion between pre-QFT-GIT period and post-QFT-GIT period. p = 0.101 comparing those with and without QFT-GIT performed in the post-QFT-GIT period; p = 0.70 comparing those with and without QFT-GIT in the pre-QFT-GIT period.

### Impact on LTBI Diagnosis and Treatment

We compared programmatic rates of LTBI diagnosis and treatment in both study periods, and examined the impact of QFT-GIT testing on diagnosis, treatment initiation, and treatment completion (Table 4). There was a significant reduction in the percentage of evaluated individuals that received a final diagnosis of LTBI in the post-QFT-GIT period (397/567 [70%]) compared to the pre-QFT-GIT period (445/452 [98%], p<0.001). In particular, in the post-QFT-GIT period, only 230/399 (58%) of those receiving QFT-GIT testing had a final diagnosis of LTBI, while 167/168 (99%) of those without QFT-GIT testing were diagnosed with LTBI on the basis of the TST result from their referring source (p<001). Among those with QFT-GIT testing conducted in the post-QFT-GIT study period, all individuals with a positive QFT-GIT had a final diagnosis of LTBI (209/209 [100%], while only 10/178 (6%) of those with a negative QFT-GIT were given a final diagnosis of LTBI on the basis of a positive TST and clinician judgment. Among these 10 QFT-GIT negative individuals, reasons given by BCHD clinicians for the diagnosis of LTBI included HIV-positivity (2), calcified granulomas or other abnormalities suggestive of TB infection on CXR (3), TST >35 m (1), young age (2), recent TST conversion within 1 year (2).

Among those given a diagnosis of LTBI, the proportions that initiated treatment were similar in the pre-QFT-GIT (341/445 [76%]) and post-QFT-GIT periods (307/397 [77%]; p = 0.81). Moreover, there was no difference in treatment initiation in the post-QFT-GIT period between those diagnosed with LTBI that had QFT-GIT testing performed (174/230 [76%]) and those that did not have QFT-GIT testing performed (133/167 [80%], p = 0.349). Among those given a diagnosis of LTBI and adequate time for treatment, the proportions completing treatment were similar between the pre-QFT-GIT period (251/445 [56%]) and the post-QFT-GIT period (174/290 [60%], p = 0.335). Treatment completion proportions, among those that initiated treatment, were also similar between the pre-QFT-GIT period (251/341[74%]) and post-QFT-GIT period (174/244 [71%]; p = 0.606). In the post-QFT-GIT period, there was no difference in treatment completion rates between those with and without QFT-GIT performed (p = 0.101). Overall, discontinuation of therapy for toxicity was similar in the pre-QFT-GIT period (7/341 (2%) and post-QFT-GIT period (8/244 (3%); p = 0.355).

## Discussion

We ascertained the clinical impact of QFT-GIT implementation in a city health department TB control program clinic, as well as barriers to its implementation. With respect to QFT-GIT uptake, we found that two-thirds of individuals referred for suspected *M. tuberculosis* infection that came for evaluation at BCHD were tested with QFT-GIT by BCHD after testing became available. Children were less likely to be tested with QFT-GIT than were older individuals. Several potential reasons include difficulty drawing blood in young children, and absence of another clinical indication for phlebotomy (i.e. liver chemistries are not routinely performed in healthy children with LTBI), and clinician discretion; in young children, clinicians at BCHD may have been more likely to accept a positive TST result in order to maximize sensitivity in this population in whom IGRA results are more difficult to interpret. In addition, individuals with HIV-infection were less likely to be tested with QFT-GIT by BCHD TB clinic, which may have represented clinician discretion in this high risk population in whom QFT-GIT has reduced sensitivity. Logistical challenges associated with QFT-GIT processing were likely the primary obstacle precluding ordering of the test for all referred individuals. QFT-GIT processing requires an initial incubation of blood at 37°C for 16–24 hours shortly after phlebotomy, after which the specimens can be stored before further processing. During the post-QFT-GIT period, this initial incubation step was performed off-site from the BCHD TB clinic. As a result, the clinic could not offer the test to individuals arriving in the late afternoon because of a need for specimen transport via courier before laboratory closure. A potential solution is to obtain an incubator for use in the TB clinic so that incubation can be initiated in the clinic immediately after phlebotomy rather than being delayed until receipt of specimens in the off-site laboratory.

Despite these challenges, there was a low proportion of test failure, with 97% of drawn QFT-GIT tests yielding interpretable results and a very low proportion of indeterminate tests (<1%). On balance, our experience suggests that implementation of QFT-GIT in local health departments is likely to be feasible, but requires attention to specimen transport schedules and/or in-clinic initiation of sample incubation, and to training of clinic staff in drawing blood from children if QFT-GIT testing of children is considered part of routine care.

Importantly, implementation of QFT-GIT testing in the BCHD TB clinic had a significant programmatic impact on LTBI diagnosis of referred individuals. In Baltimore City, individuals are referred to the BCHD TB clinic with suspected LTBI on the basis of community-based testing, usually for a positive TST. Thirty percent fewer LTBI evaluations in the BCHD TB clinic resulted in a final diagnosis of LTBI in the period after QFT-GIT implementation compared to a similar time period prior to test implementation. This reduction was driven by the additional QFT-GIT testing conducted by BCHD on LTBI referrals. In both study periods, nearly all referred individuals not receiving BCHD directed QFT-GIT testing had a final diagnosis of LTBI, with reliance on TST data from the referral source. In contrast, only 58% of individuals referred for LTBI evaluation were given a final diagnosis of LTBI if QFT-GIT was performed by BCHD as part of the diagnostic evaluation. Interestingly, while the proportion of individuals with LTBI diagnosis was reduced in the post-QFT-GIT period, there was no difference in the proportion of individuals that initiated or completed LTBI treatment between the study periods. This finding suggests that choice of diagnostic test did not influence patient behavior with regards to starting or completing treatment in our setting.

Our study evaluated factors associated with QFT-GIT positivity and offers important insights into QFT-GIT test performance and concordance with TST under operational conditions. Overall, the QFT-GIT test was positive in only half of the individuals referred to BCHD as having possible *M. tuberculosis* infection and agreed with TST results in only 52% of cases. These results suggest that TST positivity from community-based testing may have suboptimal positive predictive value for *M. tuberculosis* infection. This finding is similar to that reported in other low-prevalence settings when QFT-GIT was performed on TST positive referrals [9]. Interestingly, foreign-born individuals had higher proportions of QFT-GIT positivity (57%) compared to US-born individuals (36%) among LTBI referrals. This finding may reflect the higher risk of LTBI for foreign-born individuals from endemic settings, compared to US-born individuals. It also may speak to the technical difficulties with performing and interpreting TSTs, particularly in low-prevalence settings. Nonetheless, even among foreign-born individuals, overall QFT-GIT positivity and TST concordance were relatively low, which may indicate suboptimal TST specificity in BCG vaccinated populations. There were also differences in QFT-GIT positivity based on referral source in univariate analysis, which may represent the challenges in reliability of TST results when performed by heterogenous sources in the community.

Our study has several important limitations. Our study population consisted of individuals who were referred to the health department on the basis of prior suspicion or diagnosis of latent TB. QFT-GIT positivity and concordance with TST may differ when performed in an unselected group from the general population. Nonetheless, the BCHD TB program structure is not dissimilar from many public health TB programs in the US, and our results have relevance for urban local health departments and other facilities providing latent TB services. Without a reference standard for latent TB diagnosis, interpretation of TST and QFT-GIT discordance can be challenging [15]. Discordance, conversions, and reversions of both tests are known to occur and may be the result of intra-individual variability, timing related to TB exposure, host immunologic responses, laboratory or test procedures, or cross-reactivity with BCG. The CDC thus currently recommends that in persons with discordant test results, decisions should be made on an individual basis considering aspects that include the degree of TST induration, BCG vaccination status, quantitative QFT-GIT results, the probability of infection, and the risk of disease if infected [7]. All patients referred for evaluation at BCHD received an individual assessment based on these considerations; nonetheless our results suggest that the majority of individuals evaluated at BCHD with negative QFT-GIT results were considered to not have LTBI and were not initiated on treatment. Long term clinical outcomes in individuals with a positive TST but negative QFT-GIT are currently unknown. As such, clinical interpretations of discordant results and treatment decisions may differ in other settings based on patient risk factors or local epidemiologic considerations. While BCG vaccination status is assessed by BCHD clinicians, this data was not systematically recorded in BCHD records and thus any impact of BCG vaccination on test results could not be evaluated in this study. Our study was also limited to assessing the impact of QFT-GIT testing on LTBI diagnosis and treatment among only referred individuals. BCHD additionally performs LTBI evaluations in individuals that are close-contacts of active TB cases. To date, however, due to logistical challenges of performing phlebotomy in the field, few individuals received QFT-GIT testing as part of BCHD contact investigations, and they were not considered as part of this study. Lastly, as this study was a retrospective cohort analysis comparing two different periods of time, we cannot exclude the possibility that temporal trends could have influenced the results.

Our study also has several important strengths. In contrast to studies of QFT-GIT diagnostic accuracy, we evaluated the programmatic impact of implementing this new TB diagnostic modality in a large urban public health department under realistic operational conditions. We also report on factors associated with QFT-GIT positivity which may help guide other local health departments considering test implementation. With continued reductions in public health resources, our study has important implications for local TB prevention programs. Overall, we found that QFT-GIT implementation led to significant reductions in LTBI diagnosis and treatment at BCHD. Future studies are now needed to determine the cost-effectiveness of QFT-GIT implementation, and to examine TB reactivation rates in QFT-GIT and TST discordant patients.

## Supporting Information

Table S1 **Differences between those who did and did not come for an initial BCHD appointment among those referred for LTBI evaluation.** Abbreviations: BCHD, Baltimore City Health Department. SD, Standard Deviation *p = 0.358 comparing percentages of men and women adhering to an initial LTBI evaluation between the pre-QFT-GIT and post-QFT-GIT periods **p = 0.621 comparing mean age of individuals adhering to an initial LTBI evaluation between the pre-QFT-GIT and post-QFT-GIT periods †p = 0.917 comparing percentage of foreign-born individuals adhering to an initial LTBI evaluation between the pre-QFT-GIT and post-QFT-GIT periods.
(DOC)

Table S2 **Factors associated with QFT-GIT testing by BCHD in the post-QFT-GIT period.** †Comparison of equality of proportions receiving QFT-GIT testing among those with known ethnicities †† Comparison of equality of proportions from each referral source receiving QFT-GIT testing.
(DOC)

Table S3 **Factors associated with discordant results among those who came to BCHD for evaluation.** *excluded due to colinearity with birth country.
(DOC)
