# Supplementary material for: Programmatic Impact of QuantiFERON-TB Gold In-Tube Implementation on Latent Tuberculosis Diagnosis and Treatment in a Public Health Clinic
Source: PLoS One. 2012 May 7;7(5):e36551. doi: 10.1371/journal.pone.0036551 (PMC3346719; doi:10.1371/journal.pone.0036551)
Supplement: Table S1 — Differences between those who did and did not come for an initial BCHD appointment among those referred for LTBI evaluation. Abbreviations: BCHD, Baltimore City Health Department. SD, Standard Deviation *p = 0.358 comparing percentages of men and women adhering to an initial LTBI evaluation between the pre-QFT-GIT and post-QFT-GIT periods **p = 0.621 comparing mean age of individuals adhering to an initial LTBI evaluation between the pre-QFT-GIT and post-QFT-GIT periods †p = 0.917 comparing percentage of foreign-born individuals adhering to an initial LTBI evaluation between the pre-QFT-GIT and post-QFT-GIT periods. (DOC) [file pone.0036551.s001.doc]

Supplemental Table S1: Differences between those who did and did not come for an initial BCHD appointment among those referred for LTBI evaluation

| Characteristic |  | Pre-QFT-GIT N=607 | | Post QFT-GIT N=750 | |
| --- | --- | --- | --- | --- | --- |
|  |  | Evaluation conducted/N (%) | p | Evaluation conducted/N (%) | p |
| Number |  | 452(75%) |  | 567(76%) |  |
| Gender* | Female | 180/255 (71%) | P=0.062 | 242/325(74%) | P=0.53 |
|  | Male | 272/352(77%) |  | 325/425(77%) |  |
| Mean Age** | Evaluated by BCHD(SD) | 35.7(16) | P=0.24 | 36.2(17) | P=0.58 |
|  | No Evaluation by BCHD(SD) | 37.4(14) |  | 36.9(16) |  |
| Birth Country† | Foreign Born | 296/363(82%) | P<0.001 | 412/507(81%) | P<0.001 |
|  | Not Foreign Born | 156/244(64%) |  | 155/243(64%) |  |
| Referral source | Drug Treatment Program | 94/134(70%) | P<.001†† | 65/104(63%) | P<.001†† |
|  | Refugee | 176/194(91%) |  | 212/237(89%) |  |
|  | B-Waiver | 14/25(56%) |  | 57/72(79%) |  |
|  | Health Fairs | 17/19(89%) |  | 25/40(63%) |  |
|  | Immigration/Civil Surgeons | 18/21(86%) |  | 25/32(78%) |  |
|  | HIV | 6/7(86%) |  | 12/17(71%) |  |
|  | Local Health Departments | 31/52(60%) |  | 52/66(79%) |  |
|  | Dept of Corrections | 2/4(50%) |  | 2/3(66%) |  |
|  | Occupational Health | 10/14(71%) |  | 8/9(89%) |  |
|  | Obstetricians | 3/20(15%) |  | 8/22(36%) |  |
|  | Primary Care Provider/Other | 81/117(69%) |  | 100/148(68%) |  |

**Legend for Table S1:**

Abbreviations: BCHD, Baltimore City Health Department. SD, Standard Deviation

*p=0.358 comparing percentages of men and women adhering to an initial LTBI evaluation between the pre-QFT-GIT and post-QFT-GIT periods

**p=0.621 comparing mean age of individuals adhering to an initial LTBI evaluation between the pre-QFT-GIT and post-QFT-GIT periods

†p=0.917 comparing percentage of foreign born individuals adhering to an initial LTBI evaluation between the pre-QFT-GIT and post-QFT-GIT periods

†† Comparison of equality of proportions between referral sources adhering to an initial BCHD appointment (χ^2^ )
