# Supplementary material for: Programmatic Impact of QuantiFERON-TB Gold In-Tube Implementation on Latent Tuberculosis Diagnosis and Treatment in a Public Health Clinic
Source: PLoS One. 2012 May 7;7(5):e36551. doi: 10.1371/journal.pone.0036551 (PMC3346719; doi:10.1371/journal.pone.0036551)
Supplement: Table S2 — Factors associated with QFT-GIT testing by BCHD in the post-QFT-GIT period. †Comparison of equality of proportions receiving QFT-GIT testing among those with known ethnicities †† Comparison of equality of proportions from each referral source receiving QFT-GIT testing. (DOC) [file pone.0036551.s002.doc]

Supplemental Table S2: Factors associated with QFT-GIT testing by BCHD in the post-QFT-GIT period

| Characteristic | | Referral for LTBI | |  |
| --- | --- | --- | --- | --- |
|  |  | Total N | QFT-GIT performed by BCHD (%) | p |
| Evaluated by BCHD | | 567 |  |  |
| Eligible for BCHD QFT-GIT Testing | | 543 | 375 (69%) |  |
| Gender | Female | 234 | 169(72%) | 0.165 |
|  | Male | 309 | 206(67%) |  |
| Age | 0-2 | 3 | 0 (0%) | <.01 |
|  | 2-12 | 45 | 3(7%) |  |
|  | 13-17 | 36 | 23(64%) |  |
|  | 18-50 | 336 | 258(77%) |  |
|  | >50 | 123 | 91(74%) |  |
| Birthplace | US Born | 150 | 110 (73%) | 0.18 |
|  | Foreign Born | 393 | 265 (67%) |  |
| Ethnicity | Black | 222 | 156 (70%) | .512† |
|  | Asian/Pacific Island | 199 | 128 (64%) |  |
|  | Latino | 78 | 56 (78%) |  |
|  | White | 27 | 18 (67%) |  |
|  | Other/Unavailable | 17 | 17 (100%) |  |
| HIV | Positive | 17 | 9 (53%) | <0.01 |
|  | Negative | 388 | 305 (79%) |  |
|  | Unknown | 138 | 61 (44%) |  |
| Referral Source: | Drug Treatment Program | 65 | 47(72%) | 0.761†† |
|  | Refugee | 212 | 149(70%) |  |
|  | B-Waiver | 57 | 35(61%) |  |
|  | Health Fairs | 25 | 18(72%) |  |
|  | Immigration/Civil Surgeons | 10 | 7(70%) |  |
|  | HIV | 10 | 8(80%) |  |
|  | Local Health Departments | 51 | 37(73%) |  |
|  | Dept of Corrections | 2 | 1(50%) |  |
|  | Occupational Health | 4 | 4(100%) |  |
|  | Obstetricians | 8 | 6(75%) |  |
|  | Primary Care Provider/Other | 99 | 63(63%) |  |

**Table S2 Legend**

†Comparison of equality of proportions receiving QFT-GIT testing among those with known ethnicities

†† Comparison of equality of proportions from each referral source receiving QFT-GIT testing
