# Supplementary material for: Programmatic Impact of QuantiFERON-TB Gold In-Tube Implementation on Latent Tuberculosis Diagnosis and Treatment in a Public Health Clinic
Source: PLoS One. 2012 May 7;7(5):e36551. doi: 10.1371/journal.pone.0036551 (PMC3346719; doi:10.1371/journal.pone.0036551)
Supplement: Table S3 — Factors associated with discordant results among those who came to BCHD for evaluation. *excluded due to colinearity with birth country. (DOC) [file pone.0036551.s003.doc]

Supplemental Table S3: Factors associated with discordant results in the post-QFT-GIT period

| Characteristic | |  |  |  |  |
| --- | --- | --- | --- | --- | --- |
|  | | N | Discordant | OR | AOR |
| Both TST and interpretable QFT-GIT results available | | 352 | 169(48%) |  |  |
| Gender | Female | 162 | 82(51%) | REF | REF |
|  | Male | 190 | 87(46%) | 0.81 (.53-1.2) | .81(.5-1.3) |
| Age | 0-2 | 0 | -- | -- | -- |
|  | 2-12 | 3 | 2 (66%) | 2.3(.2-27) | 6.5(.50-82) |
|  | 13-17 | 23 | 11(48%) | 1.1(.42-2.7) | 1.8 (.62-5.0) |
|  | 18-50 | 248 | 120 (48%) | 1.1(.66-1.8) | 1.5 (.82-2.6) |
|  | >50 | 78 | 36(46%) | REF | REF |
| Birthplace | US Born | 108 | 68(63%) | REF | REF |
|  | Foreign Born | 244 | 101(41%) | **.41 (.26-.66)** | .**34 (.18-.62)** |
| Ethnicity | White | 18 | 11(61%) | REF | REF |
|  | Asian/Pacific Island | 108 | 43(40%) | .42(.15-1.2) | .82(.26-2.6) |
|  | Black | 154 | 73(47%) | .57(.21-1.6) | .72 (.25-2.1) |
|  | Latino | 56 | 31(55%) | .78(.67-2.3) | 1.4(.44-4.6) |
|  | Other/Unavailable | 16 | 11(69%) | 1.4(.34-5.8) | 1.3(.28-5.8) |
| HIV | Negative | 283 | 131 (46%) | REF | REF |
|  | Positive | 10 | 8 (80%) | 4.6 (.97-22.2) | 3.4(0.52-22.0) |
|  | Unknown | 59 | 30 (51%) | 1.2 (.68-2.1) | .79(.41-1.6) |
| Referral Source: | Drug Treatment Program | 44 | 30 (68%) | 1.3 (.58-3.0) | --* |
|  | Refugee | 144 | 52 (36%) | **.35 (.19-.64)** |  |
|  | B-Waiver | 17 | 6 (35%) | .34 (.11-1.0) |  |
|  | Health Fairs | 18 | 10 (56%) | .77(.27-2.2) |  |
|  | Immigration/Civil Surgeons | 9 | 3 (33%) | .31 (.07-1.3) |  |
|  | HIV | 8 | 7 (78%) | 2.1 (.41-11.2) |  |
|  | Local Health Departments | 35 | 14 (39%) | **.39 (.17-.91)** |  |
|  | Dept of Corrections | 1 | 1 (100%) | -- |  |
|  | Occupational Health | 5 | 2 (40%) | .41(.06-2.6) |  |
|  | Obstetricians | 6 | 5(83%) | 3.1(.34-27.8) |  |
|  | PMD/Health Centers/Other* | 63 | 39(62%) | REF |  |
| TST Induration | 0-10mm | 10 | 5(50%) | REF | REF |
|  | 10-15m | 187 | 108(64%) | 1.3(.38-4.9) | 1.62 (.35-7.6) |
|  | 15-20mm | 96 | 37(22%) | .63(.17-2.3) | .73 (0.15-3.6) |
|  | >20mm | 59 | 19(11%) | .48(.12-1.8) | .58 (0.11-3.0) |

**Legend for Table S3**

*excluded due to colinearity with birth country
